# Supplementary material for: Acceptability of COVID-19 self-testing among social and clinical vulnerable populations using a decentralized testing model in Abuja, Nigeria; A mixed methods analysis of an implementation study
Source: PLOS Glob Public Health. 2026 Jan 12;6(1):e0005679. doi: 10.1371/journal.pgph.0005679 (PMC12795379; doi:10.1371/journal.pgph.0005679)
Supplement: S1 File — (DOCX) [file pgph.0005679.s001.docx]

**S1 File. Participant Eligibility Criteria for Study Inclusion.**

Participants were eligible if they presented with any of the following symptoms:

- Fever (current or history, > 37°C),
- Sore throat
- Runny nose
- Cough
- Shortness of breath or difficulty in breathing
- Vomiting
- Nausea
- Diarrhoea
- Tiredness
- Chest pain
- Red eyes
- Loss of taste
- Loss of smell
- Headache
- Muscle or body aches
- Chill or shivering
- Difficulty speaking or confusion
- Fatigue or Dizziness
- Loss of appetite
- Abdominal pain
