# Supplementary material for: Acceptability of COVID-19 self-testing among social and clinical vulnerable populations using a decentralized testing model in Abuja, Nigeria; A mixed methods analysis of an implementation study
Source: PLOS Glob Public Health. 2026 Jan 12;6(1):e0005679. doi: 10.1371/journal.pgph.0005679 (PMC12795379; doi:10.1371/journal.pgph.0005679)
Supplement: S2 File — (DOCX) [file pgph.0005679.s002.docx]

**S2 File. List of comorbidities self-reported by study participants.** Indication of any of the following constituted clinical vulnerability.

- Aged 50 or older
- Cancer
- Kidney disease
- Lung disease, e.g. asthma
- Liver disease
- Diabetes
- Physical and Intellectual/ Learning Disabilities‡ that means you need support with daily living
- Heart conditions (such as heart failure, coronary artery disease, or cardiomyopathies),
- HIV (human immunodeficiency virus)
- Obesity (BMI ≥30 kg/m2 or ≥95th percentile in children)
- Primary Immunodeficiencies (because of medicine/ transplant/ disease)
- Pregnancy and recent pregnancy (6 weeks)
- Tuberculosis
