# Supplementary material for: Acceptability of COVID-19 self-testing among social and clinical vulnerable populations using a decentralized testing model in Abuja, Nigeria; A mixed methods analysis of an implementation study
Source: PLOS Glob Public Health. 2026 Jan 12;6(1):e0005679. doi: 10.1371/journal.pgph.0005679 (PMC12795379; doi:10.1371/journal.pgph.0005679)
Supplement: S3 File — (DOC) [file pgph.0005679.s003.DOC]

**S3 File. Participant Clinical Symptoms**

The most reported clinical symptom among participants were headache (832/1,322, 62.9%), fever (610/1,322, 46.1%), cough (573/1,322, 43.3%), and tiredness (598/1,322, 45.2%) (**Table A**)

**Table A: Reported and presenting symptoms among clients by settings**

| **Disease Presentation Characteristics** | **Total**  **N = 1322** | **PHC**  **N = 707** | **CP**  **N= 273** | **PMS**  **N = 342** |
| --- | --- | --- | --- | --- |
| Fever | 610 (46.1) | 295 (48.4%) | 89 (14.6%) | 226 (37.1%) |
| Cough | 573 (43.3) | 232 (40.5%) | 116 (20.2%) | 225 (39.3%) |
| Loss of appetite | 199 (15.1) | 74 (37.2%) | 9 (4.5%) | 116 (58.3%) |
| Loss of smell | 121 (9.2) | 59 (48.8%) | 4 (3.3%) | 58 (47.9%) |
| Tiredness | 598 (45.2) | 279 (46.7%) | 66 (11.0%) | 253 (42.3%) |
| Shortness of breath | 48 (3.6) | 28 (58.3%) | 6 (12.5%) | 14 (29.2%) |
| Runny nose | 383 (29.0) | 113 (29.6%) | 90 (23.6%) | 179 (46.9%) |
| Vomiting | 152 (11.5) | 103 (67.8%) | 15 (9.9%) | 34 (22.4%) |
| Chest Pain | 188 (14.2) | 106 (56.4%) | 25 (13.3%) | 57 (30.3%) |
| Nausea | 109 (8.3) | 49 (45.0%) | 4 (3.7%) | 56 (51.4%) |
| Diarrhoea | 93 (7.0) | 40 (43.0%) | 10 (10.8%) | 43 (46.2%) |
| Sore throat | 311 (23.5) | 123 (39.6%) | 53 (17.0%) | 135 (43.4%) |
| Headache | 832 (62.9) | 409 (49.2%) | 149 (17.9%) | 274 (32.9%) |
| Others | 61 (4.6) | 47 (77.1%) | 13 (21.3%) | 1 (1.6%) |
