# Supplementary material for: Acceptability of COVID-19 self-testing among social and clinical vulnerable populations using a decentralized testing model in Abuja, Nigeria; A mixed methods analysis of an implementation study
Source: PLOS Glob Public Health. 2026 Jan 12;6(1):e0005679. doi: 10.1371/journal.pgph.0005679 (PMC12795379; doi:10.1371/journal.pgph.0005679)
Supplement: S4 File — (DOC) [file pgph.0005679.s004.doc]

**Table A: Demographic profile of study participants who completed the IDI**

| **Variables** | **Frequency (N)** | **%** |
| --- | --- | --- |
| **Age in years** |  |  |
| 18-28 | 8 | 36.4 |
| 29-38 | 10 | 45.5 |
| 39 and above | 4 | 18.1 |
| **Gender** |  |  |
| Male | 8 | 36.4 |
| Female | 14 | 63.6 |
| **Highest Educational qualification** |  |  |
| None/Primary | 4 | 18.2 |
| Secondary | 4 | 18.2 |
| Post-Secondary | 14 | 63.6 |
| **Testing Facility** |  |  |
| PMS | 7 | 31.8 |
| CP | 7 | 31.8 |
| PHC | 8 | 36.4 |
