# Supplementary material for: Acceptability of COVID-19 self-testing among social and clinical vulnerable populations using a decentralized testing model in Abuja, Nigeria; A mixed methods analysis of an implementation study
Source: PLOS Glob Public Health. 2026 Jan 12;6(1):e0005679. doi: 10.1371/journal.pgph.0005679 (PMC12795379; doi:10.1371/journal.pgph.0005679)
Supplement: S1 Checklist — (DOC) [file pgph.0005679.s006.doc]

# Inclusivity in global research

PLOS’ policy on inclusivity in global research aims to improve transparency in the reporting of research performed outside of researchers’ own country or community and ensures that PLOS publications reporting global research adhere to high standards for research ethics and authorship. Authors of relevant research articles may be asked to complete the questionnaire below, which outlines ethical, cultural, and scientific considerations specific to inclusivity in global research. This questionnaire may be requested when researchers have travelled to a different country to conduct research, if research uses samples collected in another country, research with Indigenous populations or their lands, or if research is on cultural artefacts. Researchers travelling to another country solely to use laboratory equipment will not normally be required to complete the questionnaire. However, the questionnaire can be requested at the journal’s discretion for any submission – if you have been requested to complete this questionnaire by the PLOS journal you submitted to, please do so.

Please complete the questionnaire below and include this as a Supporting Information file with your manuscript. Note that if your paper is accepted for publication, this checklist will be published with your article in the supporting information files. Please ensure that you reference the checklist in the main body of your manuscript. We suggest adding a subsection ‘Inclusivity in global research’ to your Methods section and adding the following sentence: “Additional information regarding the ethical, cultural, and scientific considerations specific to inclusivity in global research is included in the Supporting Information (SX Checklist)”

The questions have been designed to be applicable to a wide range of study types, and there are subsections for both human subjects research and non-human subjects research. If any of the questions are not relevant to your research please mark them as “N/A” as appropriate.

**Ethical considerations, permits and authorship**

*This section is applicable to all research types.*

Provide details as to who granted permissions and/or consent for the study to take place in the Methods section of your manuscript. This should include the names of **all** ethics boards, governmental organizations, community leaders or other bodies that provided approval for the study. If individuals provided approval refer to these people by their role or title but do not list their name(s).

Reported on page number: *9 – 10 (WHO Ethical Review Committee (Protocol ID: CERC.0165), the London School of Tropical Medicine and Hygiene Intervention Research Ethics Committee (Project ID: 26886), and the Federal Capital Territory Authority (FCTA), Abuja, Nigeria Ethical Review Committee (Approval number: FHREC/2022/01/29/09-03-22))*

If there were any deviations from the study protocol after approval was obtained please provide details of these changes in the Methods section of your manuscript.

Reported on page number: N/A *(No protocol deviation recorded during the study implementation)*

Did this study involve local collaborators that are residents of the country where the research was conducted or members of the community studied? If you do not have any authors from said communities, please provide an explanation for this below.

*The study involved several local researchers from Nigeria comprising of researchers from Society for Family Health in Abuja office and Zankli Research Centre, Bingham University, Nigeria. And authors from these institutions are listed among the list of authors in the manuscript*

Everyone listed as an author should meet PLOS’ criteria for authorship and all individuals who meet these criteria should be included in the author byline, rather than the acknowledgements. For further information please see the journal’s Authorship Policy.

**Human subjects research (e.g. health research, medical research, cross-cultural psychology)**

Did you obtain written informed consent from a representative of the local community or region before the research took place? How did you establish who speaks for the community? Details of written informed consent obtained from study participants should be reported separately in the Methods section of your manuscript.

*No, written informed consent was not obtained from a community representative prior to the research. However, community entry and approval were secured through meetings with local health authorities and facility heads who supported study implementation, as well as through the implementing partner (SFH) community engagement mechanisms as part of programme delivery and design.*

How did members of the local community provide input on the aims of the research investigation, its methodology, and its anticipated outcome(s)?

*Consultations with local health facility staff, community pharmacists, and patent medicine vendors took place during the preparatory phase. Stakeholders offered insights on appropriate testing models, preferred communication strategies, and potential barriers to participation. Their feedback helped shape the study’s methodology, including site selection, participant recruitment approaches, and how to frame the benefits of self-testing. While the broader community was not directly involved in designing the research aims, the involvement of trusted local healthcare providers – alongside our implementing partner (SFH) with extensive community networks - ensured that community perspectives were considered and integrated into the study’s implementation.*

When engaging with the local community, how did you ensure that the informed consent documents and other materials could be understood by local stakeholders?

*Trained research assistants fluent in these languages provided verbal explanations and addressed questions in participants’ preferred language engaged during the study implementation. Materials were written in simple, non-technical terms and pre-tested with a small group of community members to assess clarity. This approach ensured that participants fully understood the study purpose, procedures, risks, and their rights before providing consent. Instructions for use were optimized for local languages and visual literacy.*

Will the findings of the research be made available in an understandable format to stakeholders in the community where the study was conducted (e.g. via a presentation, summary report, copies of publications, etc.)? Please provide details of how this will be achieved.

*Yes, a dissemination meeting was held in December 2023 to brief all stakeholders including government agencies, governing bodies of patent medicine vendors, pharmacist, representatives of study areas and communities, local researchers, local media, representative of study sites of the study findings and the implication for policy change in Nigeria and globally.*

**Non-human subjects research using specimens/ animals collected as part of the study, or those housed in archival collections. Examples include archaeology, paleontology, botany and zoology.**

Did the permission you obtained from a local authority to perform the study include an agreement on access to outputs and benefit sharing? This may include procedures to enable fair distribution of the benefits and resources arising from the research performed. Please include any details of Prior Informed Consent and Benefit Sharing Agreements obtained. These may be required by field-specific regulations, for example the Convention on Biological Diversity (CBD) and the associated Nagoya Protocol.

*Permission to conduct the study was obtained from relevant local health authorities, who were informed about the study objectives, methods, and expected public health value. In alignment with ethical research practices, study findings were shared with local health stakeholders through debriefing sessions, and summaries of results were made available to participating facilities to support local decision-making. While no monetary or material benefits were distributed, the study aimed to contribute to improving access to self-testing services and informing future public health strategies thereby indirectly benefiting the community.*

If the material used in your study was imported, please A) provide the year it was imported and B) indicate whether permits were obtained to import/export the materials used, C) provide details of any permits obtained. If this information is not available, please indicate this.

*SD Biosensor test kits were imported in December 2022 for the study. In compliance with Nigeria’s regulatory guidelines for the importation of in vitro diagnostic devices, an import waiver/custom clearance was obtained from the National Agency for Food and Drug Administration and Control (NAFDAC) in November 2022, prior to shipment.*

If you used archival specimens, please state how the material used in your study was acquired by the institute it is held in and provide details of any permits obtained for the original excavations/ sample collection. If this information is not available, please indicate this.

*No, archive specimen were not used for this study*

How was the potential cultural significance of the materials collected in your study to local communities considered in your research design? Were Indigenous peoples and/or local researchers and institutions involved with archaeological excavations / collection of specimens? If so, please provide a description of their involvement.

*The study did not involve the collection of culturally significant materials, archaeological excavations, or biological specimens. It focused solely on observational data related to community-based COVID-19 self-testing uptake and access.*

If your manuscript includes photographs of human remains please indicate whether authors obtained permission from descendants or affiliated cultural communities to do so.

*N/A*
