# Supplementary material for: Acceptability of COVID-19 self-testing among social and clinical vulnerable populations using a decentralized testing model in Abuja, Nigeria; A mixed methods analysis of an implementation study
Source: PLOS Glob Public Health. 2026 Jan 12;6(1):e0005679. doi: 10.1371/journal.pgph.0005679 (PMC12795379; doi:10.1371/journal.pgph.0005679)
Supplement: Attachment — Submitted filename: Response to reviewers_310725.doc [file pgph.0005679.s007.doc]

**Response to reviewers**

**PLOS Global Public Health**

31 July 2025.

Dear Dr Emma Campbell,

**Re: PGPH-D-25-00883; Acceptability of COVID-19 Self-testing among Social and Clinical Vulnerable Populations using a Decentralized Testing Model in Abuja, Nigeria; A mixed methods analysis of an implementation study.**

Thank you for facilitating the review of the above titled manuscript. Please see below a point-by-point response to each reviewers’ comments.

Please note that all line numbers refer to clean version of this manuscript.

**Responses**:

| Comment | Authors response  Manuscript amendments |
| --- | --- |
| **Journal requirements** | |
| 1. Please include a complete copy of PLOS’ questionnaire on inclusivity in global research in your revised manuscript.   Our policy for research in this area aims to improve transparency in the reporting of research performed outside of researchers’ own country or community. The policy applies to researchers who have travelled to a different country to conduct research, research with Indigenous populations or their lands, and research on cultural artefacts. The questionnaire can also be requested at the journal’s discretion for any other submissions, even if these conditions are not met. Please find more information on the policy and a link to download a blank copy of the questionnaire here: <https://journals.plos.org/globalpublichealth/s/best-practices-in-research-reporting>. Please upload a completed version of your questionnaire as Supporting Information when you resubmit your manuscript. | Thank you, this has been attached with the submission. |
| 2. In the online submission form, you indicated that [Data is available upon request from the authors.­­].   All PLOS journals now require all data underlying the findings described in their manuscript to be freely available to other researchers, either a. In a public repository, b. Within the manuscript itself, or c. Uploaded as supplementary information.  This policy applies to all data except where public deposition would breach compliance with the protocol approved by your research ethics board. If your data cannot be made publicly available for ethical or legal reasons (e.g., public availability would compromise patient privacy), please explain your reasons by return email and your exemption request will be escalated to the editor for approval. Your exemption request will be handled independently and will not hold up the peer review process, but will need to be resolved should your manuscript be accepted for publication. One of the Editorial team will then be in touch if there are any issues. | Thank you, we have uploaded the fully anonymised data into a public repository as per protocol: https://datacompass.lshtm.ac.uk.  Working repository title: Dunkley, Y (2025). STAR Africa, Asia, Americas COVID–19 preparedness (3ACP). [Collection]. London School of Hygiene & Tropical Medicine, London, United Kingdom.  Repository submission is still in review, and DOI will be shared once publicly available. |
| 3. We do not publish any copyright or trademark symbols that usually accompany proprietary names, eg (R), (C), or TM (e.g. next to drug or reagent names). Please remove all instances of trademark/copyright symbols throughout the text, including ® and ™ on page 6. | Thank you for your comment. We have deleted all trademarks within the manuscript. |
| 4. Figure 1: please (a) provide a direct link to the base layer of the map (i.e., the country or region border shape) and ensure this is also included in the figure legend; and (b) provide a link to the terms of use / license information for the base layer image or shapefile. We cannot publish proprietary or copyrighted maps (e.g. Google Maps, Mapquest) and the terms of use for your map base layer must be compatible with our CC-BY 4.0 license.   Note: if you created the map in a software program like R or ArcGIS, please locate and indicate the source of the basemap shapefile onto which data has been plotted.  If your map was obtained from a copyrighted source please amend the figure so that the base map used is from an openly available source. Alternatively, please provide explicit written permission from the copyright holder granting you the right to publish the material under our CC-BY 4.0 license.  Please note that the following CC BY licenses are compatible with PLOS license: CC BY 4.0, CC BY 2.0 and CC BY 3.0, meanwhile such licenses as CC BY-ND 3.0 and others are not compatible due to additional restrictions.  If you are unsure whether you can use a map or not, please do reach out and we will be able to help you. The following websites are good examples of where you can source open access or public domain maps: * U.S. Geological Survey (USGS) - All maps are in the public domain. ([http://www.usgs.gov](http://www.usgs.gov/)) * PlaniGlobe - All maps are published under a Creative Commons license so please cite “PlaniGlobe, [http://www.planiglobe.com](http://www.planiglobe.com/), CC BY 2.0” in the image credit after the caption. (<http://www.planiglobe.com/?lang=enl>) * Natural Earth - All maps are public domain. ([http://www.naturalearthdata.com/about/terms-of-use/](http://www.naturalearthdata.com/about/terms-of-use)) | We created the map in QGIS using openly available administrative boundary shapefiles from the GADM (Global Administrative Areas) database, available at: <https://gadm.org/download_country.html> (select Nigeria).  The GADM licence agreement states that the data are freely available for academic use and other non-commercial use, and that “you can use the maps you made with GADM data for figures in articles published by PLoS”. The agreement can be found here: <https://gadm.org/license.html> |
| **Reviewer #1** | |
| 1. This is a mixed-methods study exploring de-centralised and self-testing for COVID-19 in Abuja, Nigeria. The qualitative findings provide useful insights into the reasons why people prefer to access testing at different types of providers and into how testing can be expanded to those with limited access. The study would benefit by more detail on how participants were selected for the qualitative component and a more in-depth discussion on the significance of these findings for conditions other than COVID-19. More specific comments below. | Thank you, these two points are addressed in their respective sections below. |
| 2. Lines 70-71 “Diagnostic testing plays a critical role in managing outbreaks like COVID-19 by enabling patient management and isolating positive cases to reduce mortality” – please rephrase as the direct aim of isolation is not reducing mortality but transmission (reduced mortality is a later consequence of decreased transmission) | Thank you for your comment, the sentence has been rephrased.  “*Diagnostic testing can play a critical role in managing outbreaks like COVID-19 through enabling patient management and isolating positive cases to reduce transmission, and associated mortality*.”  Lines 57-9. |
| 3. Line 75 “Nigeria CDC introduced antigen-based rapid diagnostic tests (Ag-RDT) in 2020” – specify in which month rapid testing was introduced | The month is now included  “*The Nigeria CDC introduced antigen-based rapid diagnostic tests (Ag-RDT) in November 2020*.”  Lines 62-3. |
| 4. Line 117: describe in more detail in the methods how the random collection of participants for the interviews was conducted. Were the interviews conducted on the same day as the testing? | We have provided additional detail on participant selection for the qualitative interviews – confirming this was purposive rather than random:  “*A subset of participants were purposively sub-sampled and engaged for qualitative IDI following testing, based on age, gender, educational status and testing facility*.”  Lines 118-120. |
| 5. Table 2: explain what “Household feeding concern means” and the reason why the proportion of those with feeding concerns is quite different than of those in the poorest stratum of wealth (29.7% vs. 4.5%) | **Thank you for your comments. “Household feeding concerns” has been defined as a footnote in Table 2.**  ***“Perceived Household Feeding Concerns refers to a situation where a participant reports being worried or anxious about their households ability to provide enough food for its members a week prior the study”***  The reason why the proportion of those with feeding concerns is quite different than of those in the poorest stratum of wealth (29.7% vs. 4.5%) is interesting; both measures are self-reported perceptions, albeit of different constructs (one of poverty and the other of household food security). It may be that perceived poverty is a more stigmatising variable than perceived food security and thereby underreported.  It may also be that perceived food insecurity also reflects food availability within the country, not simply a measure of poverty. However, this is author conjecture and as such not included within the main manuscript. |
| 6. Line 184: it would be interesting to know why the percentages of women attending the different types of facilities were different and discuss the differences in income and attendance to the different types of facilities. | The gender and wealth differences in attendance at the different facilities is interesting, and we have explored the implications of this in the discussion –  *“A higher proportion of participants from CP identified as being in the poorest wealth category, potentially representative of the location of CP in more urban settings where income inequality may be starker than rural locations. PMS models also reached more men; a group generally underrepresented in health service provision.”*  Lines 264-7. |
| 7. Table 3: would the authors be able to expand the categories for the number of comorbidities with an additional stratum. This would be to better understand how many participants had multiple co-morbidities (while excluding older age and pregnancy) | We have expanded the categories of comorbidities however we were not able to exclude older age and pregnancy as at the point of data collection the specific comorbidity was not named; rather the number of comorbidities was recorded. |
| 8. Line 220 (or at the beginning of the results section) report on the numbers approached for IDI and on how many refused to be interviewed | We have included the total number of clients approached per study facility.  “*22 of 45 participants approached for IDI agreed to participate: PHC (8/22, 36.3%), CP (7/22, 31.8%) and PMS (7/22, 31.8%).”*  Lines 212-213. |
| 9. Table 5: clarify whether the logistic regression analysis was adjusted or unadjusted (I assume unadjusted) | Thank you. Yes, the logistic regression analysis is unadjusted (univariate analysis).  *“We examined associations between social and clinical vulnerability factors and primary outcomes at study settings using unadjusted logistic regression”*  Lines 153-5. |
| 10. Lines 379-385: the sentence is very long, please break down to improve readability | Thank you – the broader discussion has been rephrased for clarity, with this sentence removed. |
| 11. Add some context to better understand the low positivity during the study period – was the study conducted during a nationwide Covid-19 “wave” or during a low-intensity transmission period? | Thank you – this is an important point –  “*Although a higher proportion of clinically vulnerable clients utilized PHC outlets for testing, test positivity rates remained low throughout reflecting the implementation of this study during a period of low community transmission towards the end of the COVID-19 pandemic.*”  Lines 268-70. |
| 12. Discuss in more detail how the findings from this study could be applied for scaling up and improving access to testing for other conditions (e.g future perspectives) | Thank you, we have considered this important point in the discussion through the following:  *“The high uptake and acceptability of COVID-19 self-testing through CP and PMS models in Nigeria demonstrates a promising, scalable and equitable approach for improving access to testing for other conditions, including, potentially, pandemic-potential pathogens. Leveraging these community-based outlets, especially PMS, which effectively reaches socially vulnerable populations can theoretically enhance early diagnosis, reduce healthcare access barriers, and support decentralized public health strategies. Self-testing technologies can further decentralize diagnostics into the home, aligning with expressed user preferences. More research is needed on the uptake and acceptability of these models and technologies in periods of acute disease transmission and for other emerging disease conditions. How this is taken forward and integrated into public health programming is a key next step.”*  Lines 284-93. |
| 13. Revise spelling as there are some minor typographical errors (e.g. missing plurals) | Thank you, we have conducted a substantial proof-read to resolve copy errors. |
| **Reviewer #2** | |
| 1. This is great research to help demonstrate the use of self-testing for transmissible diseases and utilize a mixed methods model to understand the potential benefits and acceptability of these tests. | Thank you for your comments. |
| 2. Introduction: This introduction set the context up well for readers to understand the gaps and objectives of this project. I would have liked to see more about how this study relates to other research out there around the acceptability of self-testing or perhaps other health outreach models that showed improvement when expanding to these more community-based clinic settings. | Thank you for your comment. The introduction has been rewritten (see reviewer #3 for rationale)  More context has been included:   - On previous studies reporting on the use of PMS and CP to deliver health services in the Nigerian context - On evidence generated from decentralization of diagnostics through self-testing from other disease areas (HIV)   *“Global evidence from decentralized HIV testing service delivery models have demonstrated reductions in structural barriers and expanded reach among underserved populations [16-17]. Decentralizing testing sites and technologies, and moving them into communities can increase accessibility, through reducing structural barriers such as travel distance, waiting times, and stigma for vulnerable populations who may otherwise not access services [12].*  *In Nigeria, PMS and CP have increasingly been leveraged to deliver essential health services in vulnerable communities particularly in areas with limited access to formal healthcare [18-30]. PMS have been documented as the first point of care for many Nigerians, especially in rural areas, providing treatment for malaria, diarrhea, pneumonia, and family planning services for both children and adults. Decentralized malaria rapid diagnostic tests and treatment initiations in CP and PMS have improved malaria case detection and treatment accuracy in Lagos [29]. CP have also been explored as delivery models for HIV pre-exposure prophylaxis (PrEP) and are recognized for their role in integrating family planning with HIV services in resource-limited urban settings [31].”*  Lines 76-90. |
| 3. The final paragraph states that the testing at the CP and PMS will be compared to the provider delivered testing at the PHCs but I don’t see that concept again throughout the rest of the manuscript leading me to wonder what role the PHCs had in this study. | Thank you – this is an important point.  We have refined the study aims (as per Reviewer #3) to the following:  “*this study aims to evaluate the uptake of decentralized COVID-19 testing models, and the acceptability of decentralized COVID-19 self-testing models among socially and clinically vulnerable populations in Abuja.*”  Lines 92-4.  We have also therefore restructured the methods to be clearer about the role that PHCs play as comparators for uptake of decentralised testing models:  *“The primary outcome was the difference in uptake of COVID-19 testing interventions – including self-testing - by facility, defined as the percentage of eligible clients who accepted COVID-19 Ag-RDT testing when offered across PHC, CP, and PMS settings. The secondary outcome was the acceptability of COVID-19 self-testing measured as the percentage of eligible clients who opted for self-testing over provider-delivered testing in PMS and CPs.”*  Lines 137-41. |
| 4. Methods: There are a few areas in the methods that I do not feel were described in sufficient detail to be reproducible. Please add rationale or a citation for why you selected the symptom requirements you did for eligibility. | Thank you for your comments. The rationale for selecting these symptoms to assess eligibility is now included:  *“The inclusion of these symptoms as eligibility criteria for this study was based on their documented association with COVID-19 across multiple clinical and epidemiological studies”*  Lines 106-108. |
| 5. Figure 2 should be redone. It is blurry and not readable. | Thank you, we have resubmitted the figure after using the recommended PACE software. Please advise if this is better, otherwise we will reach out directly to the editors to discuss best next steps. |
| 6. I’d like to see more details on the data collection: were the socioeconomic indicators self-reported or did you use a validated questionnaire? Please describe in more detail and/or add a citation. It is also unclear to me what data were collected through medical records, and which were self-reported or through the participant log. | Thank you, we have expanded the data collection section.  *“Data was collected through structured quantitative survey-tools and semi-structured qualitative interviews. A participant log and structured questionnaire were used to obtain demographic data, including sex, age, and education level, along with socioeconomic indicators such as food security status and literacy. Socioeconomic indicators were self-reported by participants using questions from previously validated tools (the Household Food Insecurity Access Scale (HFIAS) and national literacy assessment items) [38]. Information on COVID-19 symptoms, perceived severity, risk perception, vaccination status, prior hospitalization due to COVID-19, testing preferences (self-testing vs. provider-administered testing), and testing outcomes were also collected. Clinical history was self-reported by participants unless otherwise documented in accompanying referral slips or health cards.”*  Lines 122-131. |
| 7. I’d also like to see more details on how participants were randomly selected for the interviews. Were there any considerations for balanced sex, age, location to ensure a balanced group. Was saturation determined? | Thank you, we have removed comment on random selection and clarified this was purposive recruitment across age, gender, education and facility.  *“A subset of participants were purposively sub-sampled and engaged for qualitative IDI following testing, based on age, gender, educational status and testing facility.”*  Lines 118-120. |
| 8. Please describe in the methods if the surveys and interviews were conducted in English or other languages? | Thank you - this is now included.  *“Surveys and interviews were conducted in English, Pidgin or Hausa, depending on participant preference. Trained multilingual interviewers facilitated this process.”*  Lines 133-35. |
| 9. The limitations state the instructions for the self-test were in English, please describe if language or literacy was also an eligibility requirement. | Thank you – we have clarified this:  *“There were no literacy or language eligibility requirements.”*  Line 108. |
| 10. Results: Quantitative results are straightforward. For the interviews, please add how many were invited to participate in interviews and describe the demographics of the population that agreed/completed the interviews. | Thank you – we have included these as a new supplement to save space (S4. Demographics of IDI participants.) |
| 11. I appreciate the themes discussed in the qualitative results but do feel like sections are redundant and could be written in a more concise manner. | Thank you – this is an important point and again reflects reviewer #3, this has been substantially copy-edited and condensed. |
| 12. Miscellaneous: I got the impression various sections were written by different authors and could use a review by the first author to ensure consistency in formatting and language. | Thank you – this has been actioned throughout. |
| 13. There are multiple instances of re-defining acronyms that have already been defined (e.g., “patent medicine stores (PMS)”) – define the first time then use PMS throughout. | Thank you – this has been actioned throughout. |
| 14. Also, one of the qualitative themes has the quotes in the paragraph where others have quotes as their own paragraph. | Thank you, the quote paragraphing is now consistent throughout. |
| 15. I think this paper could use more editing to be more concise, avoid redundancies, and run on sentences. | Thank you, the total paper now stands at less than 3500 words. |
| **Reviewer #3** | |
| In their study “Acceptability of COVID-19 Self-testing among Social and Clinical Vulnerable Populations using a Decentralized Testing Model in Abuja, Nigeria; A mixed methods analysis of an implementation study” Isere et al. assess the acceptability of as well as the advantages and challenges when implementing COVID-19 self-testing through different modes of care. Overall, I find the study very well conducted and results laid out clearly.  However, I have two comments concerning (1) the focus and novelty of results and (2) the length of the manuscript and abstract, which are further outlined below.  The overall focus of the manuscript is not yet clear to me. Based on its current version, I believe the authors concentrated on three questions: **first, to assess the general uptake of Ag-RDT testing for SARS-CoV-2 (see line 134); second, to evaluate the general acceptability of SARS-CoV-2 self-testing (see line 135); third, to analyze differences in uptake and acceptability between centralized versus decentralized test distribution (i.e., between community pharmacies and patent medicine stores versus primary health centers).**  The first and second question have already been discussed widely in scientific literature. The third question, however, has not yet been evaluated thoroughly and answering it could provide novel perspectives on implementing antigen (self-)testing for SARS-CoV-2.   1. Therefore, I would suggest to refine the manuscript’s **introduction, presentation of results, and discussion**, to focus **only on evaluating the difference in testing uptake and acceptability between the different modes of care** (of note, I don’t expect the authors to change the analysis underlying their manuscript or to make larger adjustments in the results section – I believe the analysis and results already entail all relevant aspects to assess differences in participation in SARS-CoV-2 antigen testing between different modes of test distribution). **Specifically, the authors could adjust the title of the manuscript, e.g., along the lines of “Centralized versus decentralized test distribution models to enhance COVID-19 antigen testing participation in vulnerable populations: a mixed-methods study”.** | Thank you – this is a very helpful review – and we have amended the introduction, methods, results and discussion as per your comments to better streamline the paper to reflect the focus on evaluating the difference in testing uptake and acceptability of different models of COVID-19 test between the different modes of care.  We have however not at this stage changed the study title – as we do want to ensure that we retain emphasis on self-testing as a vehicle of decentralisation; we feel that preferences relating to self-testing within decentralised settings represents an important additional narrative to enhance the discussion around decentralisation for access for vulnerable populations.  We hope that the amended study aim better reflects this: “*this study aims to evaluate the uptake of decentralized COVID-19 testing models, and the acceptability of decentralized COVID-19 self-testing models among socially and clinically vulnerable populations in Abuja.*”  Lines 92-4. |
| 2. Furthermore, in the introduction, I would suggest removing line 71 (“In Nigeria, …”) to 77 (“… self-testing.”). | Thank you – this has been removed. |
| 3. Building on what is already stated in line 78 to 85, the freed space could be used to **highlight clearly why vulnerable populations in Nigeria are disadvantaged by centralized modes of test distribution and how these disadvantages could be resolved through decentralized testing.** | The introduction has been rewritten as per your suggestions to highlight clearly why vulnerable populations in Nigeria are disadvantaged by centralised models of test distribution, as well as the relative benefits of decentralisation – both in terms of facility type but also in terms of self-testing as a technology:  *“Centralized testing models disproportionately affect socially vulnerable groups, including those living in rural areas, individuals with limited financial means, and people with underlying health conditions, who often face additional barriers to accessing care [12]. Rural communities throughout Nigeria routinely face greater healthcare disparities, given remote geographies, poorer infrastructure, and higher poverty rates which can limit access to testing and treatment [13]. The economic shocks of COVID‑19 amplified vulnerabilities: lockdowns and inflated costs fell hardest on low‑income and informal sector families, deepening exclusion from centralized services [14-15]. Existing inequities heighten the clinical risk of undiagnosed disease among these vulnerable populations.*  *Decentralized testing models explored in Nigeria included distribution of self-tests through Community Pharmacies (CP) and Patent Medicine Stores (PMS); these offer a promising solution to the limitations of centralized testing facilities. Global evidence from decentralized HIV testing service delivery models have demonstrated reductions in structural barriers and expanded reach among underserved populations [16-17]. Decentralizing testing sites and technologies moving them into communities can increase accessibility, through reducing structural barriers such as travel distance, waiting times, and stigma for vulnerable populations who may otherwise not access services [12].”*  Lines 65-81. |
| 4. In the methods, line 134-135, I would suggest to define the *difference* in uptake and acceptability as the primary and secondary outcome. | Thank you, we have refined the outcomes to better reflect the study aims:  *“The primary outcome was the difference in uptake of COVID-19 testing interventions – including self-testing - by facility, defined as the percentage of eligible clients who accepted COVID-19 Ag-RDT testing when offered across PHC, CP, and PMS settings. The secondary outcome was the acceptability of COVID-19 self-testing measured as the percentage of eligible clients who opted for self-testing over provider-delivered testing in PMS and CPs.*  Lines 137-141. |
| 5. In **the results section and discussion**, I would suggest to remove all text not specifically related to assessing the difference between centralized and decentralized testing (see my second comment). | Thank you for this comment – we have restricted the analysis presented and discussed to the difference in uptake and acceptability outcomes as per your recommendations.  This entails:   - Moved table 1 (clinical presentation) to a Supplement (Supplement 3). - Condensed the qualitative results section and clearly demarcated how the results align with the two outcomes (uptake and acceptability) - Removed table 4 - retaining relevant points in the narrative (nothing additional in the table that was not already described in the text). - Condensed the discussion substantially |
| 6. To enhance readability, I would suggest to shorten the manuscript on several instances. First, I would ask to limit the abstract to a maximum of 350 words. | Thank you for this comment, the abstract has been reduced to less than 350 words. |
| 7. Secondly, when presenting the main characteristics of the study population in lines 171 to 197, I would suggest to focus the text on those 5-6 characteristics of the study participants that are most essential for understanding the study’s conclusion. | All characteristics referred to in the study are now related to the concluding points raised in the discussion. |
| 8. Thirdly, I would suggest to move the section on ‘Preference for self-test over professional delivered testing’ to the Supplements, aside from those parts of this section that specifically add to understanding the impact of centralized versus decentralized test distribution models (see my first comment). | Thank you – I hope our response to point 3. above has demonstrated the rationale for retaining the preference for self-test (as a decentralised technology) as part of the qualitative results.  This section has however been substantially reduced. |
| 9. Similarly, in the discussion, I would suggest to remove lines 343-370 and lines 389-395 except those parts that concern the differences in centralized versus decentralized test distribution models. | Thank you – we have now conducted a substantial rewrite of the discussion – as also requested by reviewer #1 and #2 comments to streamline. |

Yours sincerely,

Yasmin Dunkley
