# Supplementary material for: Acceptability of COVID-19 self-testing among social and clinical vulnerable populations using a decentralized testing model in Abuja, Nigeria; A mixed methods analysis of an implementation study
Source: PLOS Glob Public Health. 2026 Jan 12;6(1):e0005679. doi: 10.1371/journal.pgph.0005679 (PMC12795379; doi:10.1371/journal.pgph.0005679)
Supplement: Attachment — Submitted filename: Response to reviewers_100925.doc [file pgph.0005679.s008.doc]

**Response to reviewers**

**PLOS Global Public Health**

10 September 2025.

Dear Dr Shifa S. Habib,

**Re: PGPH-D-25-00883; Acceptability of COVID-19 Self-testing among Social and Clinical Vulnerable Populations using a Decentralized Testing Model in Abuja, Nigeria; A mixed methods analysis of an implementation study.**

Thank you for facilitating the review of the above titled manuscript. Please see below a point-by-point response to each reviewers’ comments.

Please note that all line numbers refer to clean version of this manuscript.

In addition to the revisions below, we re-uploaded supplement 4 as we noticed a small typographical error stating 18 IDI male participants rather than 8.

**Responses**:

| Comment | Authors response  Manuscript amendments |
| --- | --- |
| **Reviewer #3** | |
| I would like to thank their authors for the thorough implementation of the reviewers’ comments. Besides four very minor suggestions (see below), I don’t have any further comments.   1. - Please add the link (https://datacompass.lshtm.ac.uk.) to the online data repository to the main manuscript. This could either be done through a ‘Data availability’ statement at the end of the manuscript, or by including mentioning it in the methods section. | Thank you for your comprehensive review. We have now added the link to a data availability statement at the manuscript end. |
| 2. Line 78 to 81: Please revisit this sentence, as it seems to be missing words. | Thank you, we have revised this sentence.  *“Decentralizing testing sites and technologies can increase accessibility, through reducing structural barriers such as travel distance, waiting times, and stigma for vulnerable populations who may otherwise not access services [12].*” |
| 3. Line 288: I would suggest to add a comma after ‚populations’. Please also check the remaining manuscript for any potential typographical inconsistencies. | Thank you we have added this comma and checked for remaining typographical inconsistencies. |
| 4. Line 318: Please complete the section 'Author contributions' | Thank you, this is now completed. |

Yours sincerely,

Yasmin Dunkley
